# Spatiotemporal distribution and determinants of overweight or obesity among urban women in Ethiopia: a multivariate decomposition analysis

**DOI:** 10.1186/s12905-022-02102-4

**Published:** 2022-12-05

**Authors:** Melkalem Mamuye Azanaw, Edgeit Abebe Zewde, Alemayehu Digssie Gebremariam, Fentaw Teshome Dagnaw, Dessalegn Tesfa Asnakew, Ermias Sisay Chanie, Dejen Getaneh Feleke, Sofonyas Abebaw Tiruneh

**Affiliations:** 1 Department of Public Health, College of Health Sciences, Debre Tabor University, Debre Tabor, Ethiopia; 2 Department of Biomedical Sciences, College of Health Sciences, Debre Tabor University, Debre Tabor, Ethiopia; 3 Department of Pediatrics Nursing, College of Health Sciences, Debre Tabor University, Debre Tabor, Ethiopia

**Keywords:** Trends, Overweight, Obesity, Urban women, Decomposition analysis, Ethiopia

## Abstract

**Background:**

Overweight /obesity is a global public health concern. It is higher among women than men in most continents of the world. This study aimed to determine the spatiotemporal distribution and determinants of changes in overweight/obesity over time among urban women in Ethiopia.

**Methods:**

We used data from three consecutive Demographic and Health Surveys in Ethiopia (2005, 2011, and 2016). The total weighted sample of 1112 in 2005, 3569 in 2011, and 3071 in 2016 urban women were included in the analysis. The primary outcome measure of this study was the spatiotemporal distribution and trends over time in overweight/obesity. Factors contributing to change in overweight/obesity were examined using a logit-based multivariate decomposition analysis.

**Results:**

Overweight/obesity increased from 14.2% in 2005 to 21% in 2016. Approximately 61.3% of the overall increase in overweight/obesity among urban women was due to the difference in coefficient (difference in the effect of characteristics) across the surveys. Changes in the composition of women aged 25–49 years (β = 0.012, 95% CI 0.008, 0.015), married women (β = 0.010, 95% CI 0.006, 0.014), women with formal education (primary: β = 0.007, 95% CI 0.003, 0.011, higher education: β = 0.014, 95% CI 0.006, 0.022), women with formal employment (β = 0.006, 95% CI 0.001, 0.011), and women with informal employment (β = − 0.002, 95% CI − 0.003, − 0.0004) were factors contributing to the change in overweight/obesity from 2005 to 2016. The risk difference (RD) in women’s overweight/obesity significantly varied across regions in urban Ethiopia. Furthermore, a high proportion of overweight/obesity was found mainly in Tigray, Oromia, Amhara, and Addis Ababa.

**Conclusions:**

The rate of overweight/obesity among women in urban Ethiopia has shown a significant increase over the last 11 years. This rate change was due to changes in the composition of women’s age, educational status, marital status, and employment status. Therefore, program interventions should be targeted at older (> 25 years), educated, married, Addis Ababa residents, and formally employed women.

## Background

Overweight/obesity denotes increased adiposity and expansion of visceral fat which is the initiator of insulin resistance, oxidative stress, and chronic inflammation [1]. The world health organization defines overweight/obesity as excessive fat accumulation which poses a risk to health [2]. Women´s nutritional status was measured by body mass index (BMI). The BMI defined as weight in kilograms divided by height in meters squared (kg/m^2^) was used to measure overweight and obesity. A BMI of above 24.99 kg/m^2^ was used as the cut-off for overweight while BMI above 29.99 kg/m^2^ was used to indicate obesity. Individuals with either overweight or obese were combined into one category and coded as “1” and others were coded as “0” [3]. Being overweight and obese is becoming a global public health concern. It is higher among women than men in most continents of the world [4] According to the 2021 global nutrition report, 2.2 billion adults are overweight or obese. Of these 772 million are affected by obesity and 40.8% of overweight adults are women of reproductive age [3, 4]. Sex-specific biological differences and gender disparity with social, cultural, and occupational factors contribute to increased obesity in women [5, 6].

Overweight or obesity among women worldwide has increased from 31.7% (609.8 million) in 2000 to 39.2% (1.02 billion) in 2016 [7]. Although the prevalence of overweight and obesity is higher in the developed world, the trend is increasing in the less developed world mainly in South Asia, and Africa [8]. Nutrition transition with the adoption of a culture of increased consumption of energy-rich food with a high level of sugar and saturated fat combined with a lack of physical activity contributes to the increasing trend of overweight/obesity in the low-income world [9]. In many African countries, overweight/obesity has doubled since 1991[10]. Although Ethiopia has one of the lowest prevalence of overweight/obesity in Africa, it is no different from the others since its prevalence increased from 10.9% in 2000 to 21.4% in 2016[11].

Being overweight/obese increases the risk of hormonal irregularities, infertility [12], breast cancer [13, 14], and adverse maternal and neonatal outcomes in women [15]. Being overweight/obese is a common risk factor for non-communicable diseases including type II diabetes mellitus, coronary heart disease, and cancer [16]. Although obesity poses a health risk for both sexes, women face greater metabolic risks and increased mortality from chronic diseases [17]. Biological pathways including menopause-related cardiovascular changes such as hyperandrogenism increased insulin resistance, dyslipidemia, and gender-related social and cultural behaviors contribute to higher risks of mortality and morbidity seen in women [18, 19].

Different factors predispose women to overweight/obesity including being post-menopausal [20–22], older age [23], use of hormonal contraceptives [24, 25], being urban residents, and having a higher level of education [11, 26], having heavy alcohol drinking [27, 28], rich wealth index [26, 29], being married [29], and multiparity [29, 30].

There is a variation in the determinants of overweight/obesity in Ethiopian regions. Although different studies have attempted to identify the predisposing factors for overweight/obesity in Ethiopia, the decomposition analysis of overweight/obesity has not been performed among urban women. Moreover, identifying hot spot regions of Ethiopia for overweight/obesity using spatial clustering help with specific interventions. Therefore, this study fills the gap by showing the trend of overweight/obese women of all reproductive age groups. Identifying the contributing factors of overweight/obesity among women in urban Ethiopia can help health planners and policymakers with specific interventions to decrease overweight/obesity.

Based on the aforementioned rationale, this study aimed to determine the trends of overweight/obesity and its spatiotemporal distributions using the previous consecutive National Demographic and Health Survey data (2005, 2011, and 2016).

## Methods

### Study design, area, and period

The nationally representative repeated cross-sectional study design was employed using the 2005, 2011, and 2016 Ethiopian Demographic and Health Surveys (EDHS). The surveys are nationally representative household surveys that collect a very wide range of population, health, and other important indicators covering all 9 regions namely, Tigray, Afar, Amhara, Benishangul-Gumuz, Gambela, Harari, Oromia, Somali, and Southern Nations Nationalities and Peoples of Region and two city administrations (Addis Ababa and Dire Dawa) in Ethiopia. Ethiopia lies between latitudes 3° and 14° N and longitudes 33° and 48° E in the horn of Africa.

### Data source and study population

The data were accessed from the official database of the DHS program (www.majordhsprogram) after permission was granted through an online request by explaining the objective of our study. The source population was women of reproductive age who gave birth 5 years before each survey in Ethiopia. Our data are restricted to women living in urban places of residence. In each survey, a nationally representative sample of 1112 in 2005, 3569 in 2011, and 3071 in 2016 weighted number of women participated.

### Data collection tools and procedures

Data were collected in two stages for each survey year. Stratification was also made based on the place of residence in each region of the country. However, the current study incorporated only urban dwellers. This study included EAs of 145 in 2005, 187 in 2011, and 202 in 2016. In the second stage, a fixed number of households were selected in each EA for each survey using systematic sampling. The detailed sampling procedure is available from the EDHS reports on the Measure DHS website www.messdhs.com for each survey.

### Study variables

#### Dependent variable

The dependent variable was overweight or obese, categorized dichotomously as “Yes / No”.

#### Independent variables

The independent variables were socio-demographic, health facility, behavioral-related, and community-level variables in the three consecutive surveys.

### Statistical analysis

The data were cleaned and analyzed using the STATA version 16 software. The sample weight was determined for further analysis. The data were weighted using cluster number, primary sampling unit, and strata before any statistical analysis to restore the representativeness of the survey and to tell the STATA to consider the sampling design when calculating SEs, as the total sampling distribution looks like the country’s actual population distribution.

Descriptive, trends over time, associated factors, and decomposition analysis of overweight/obesity were performed. Data from three EDHS surveys were appended into two and formed three data sets (2005–2011, 2011–2016, and 2005–2016 datasets) after extracting explanatory variables for each study phase for decomposition analysis. However, we only used datasets from 2005 to 2016 because it incorporates information from the other datasets.

A non-linear multivariate logit decomposition model was used to identify the factors that contributed to the change in the overweight/obesity rate over the last decade. The decomposition analysis has identified the sources of changes in the overweight/obesity rate over the last 11 years.

The output from the multivariate decomposition logistic regression analysis had two contribution effects. These effects were the compositional differences (endowments) ‘E’ and the effects of characteristics which are the difference in the coefficients or behavioral change ‘C’ responses for the selected predictor variables. In the non-linear model, the dependent variable is a function of a linear combination of predictors and regression coefficients:

For logistic regression, the Logit or log-odd of overweight/obesity is taken as:

The component labeled ‘E’ refers to the part of the differential attributable to differences in endowments or characteristics (explained component). The ‘C’ component refers to the part of the differential attributable to differences in coefficients or effects (unexplained component) [31].

The equation can be presented as:

$$Logit(A) - Logit(B) = [\beta 0A - \beta 0B] + \sum {XijB*} [\beta ijA - \beta ijB] + \sum {\beta ijB*} [XijA - XijB]$$

XijB is the proportion of the jth category of the ith determinant in the DHS 2005, XijA is the proportion of the jth category of the ith determinant in the DHS 2016, βijB is the coefficient of the jth category of the ith determinant in the DHS 2005, βijA is the coefficient of the jth category of the ith determinant in the DHS 2016, β0B is the intercept of the regression equation fitted to the DHS 2005, and β0A is the intercept of the regression equation fitted to the DHS 2016.

The recently developed multivariate decomposition for the non-linear model was used for the decomposition analysis of overweight/obesity using *mvdcmp* STATA command.

### Spatial analysis

#### Spatial clustering

The weighted prevalence in each survey was mapped to illustrate the distribution of overweight/obesity in urban Ethiopia in ArcMap. The spatial autocorrelation (Global Moran’s I) statistic was used to measure the overweight/obesity patterns in the study area. A statistically significant Moran’s I (*p* < 0.05) was used as an indicator of spatial autocorrelation.

#### Spatial interpolation

A Spatial interpolation technique was used to predict overweight/obesity of the un-sampled areas in the country based on sampled EAs using Ordinary Kriging spatial interpolation methods.

#### Spatial scan statistical analysis

Spatial scan statistical analysis was employed to test for the presence of statistically significant spatial clusters of overweight/obesity using Kuldorff’s SaT Scan version 9.6 software. The spatial scan statistic uses a circular scanning window that moves across the study area.

Overweight/obese women were considered as cases and those who had no overweight/obesity as controls fit the Bernoulli model. The number of cases in each location had a Bernoulli distribution and the model required data for cases, controls, and geographic coordinates. The default maximum spatial cluster size of < 50% of the population was used, as an upper limit, which allowed both small and large clusters to be detected and ignored clusters that contained more than the maximum limit. For each potential cluster, a likelihood ratio test statistic was used to determine if the number of observed overweight/obese individuals within the potential cluster was significantly higher than expected or not. The primary, secondary and tertiary clusters were identified and assigned *p* values and ranked based on their likelihood ratio test, based on 999 Monte Carlo replications.

## Results

### Characteristics of study participants

A total of 1112, 3569, and 3071 urban women were interviewed in the 2005, 2011, and 2016 survey years, respectively. The mean (± SD) age of respondents in 2005, 2011, and 2016 surveys were 26.4 (± 9.3), 26.3 (± 8.8), and 27.4 (± 8.9) years, respectively. From 2005 to 2016, the proportion of women with formal education increased from 76.7% to 83.7%. The proportion of women with higher education increased from 6.8% to 21.0%. The proportion of married women increased from 36.3% in 2005 to 44.9% in the 2016 survey whereas the proportion of never-married women declined from 46.7% in 2005 to 41.5% in 2016. The proportion of women who used contraceptive methods increased by 9.6%from 2005 to 2016. The proportion of women who smoked cigarettes increased from 7.2% in 2005 to 30.7% in 2016. On the other hand, the proportion of improved water sources at the household level increased from 50.8% in 2005 to 78.7% in 2016 (Table 1).

**Table 1:** Respondents’ distribution according to sociodemographic and behavioral variables during 2005–2016 in Ethiopia

Variables	Survey year		
	EDHS-2005 N (%)	EDHS-2011 N (%)	EDHS-2016 N (%)
Number of women (weighted)	1112	3569	3071
Sex of household headed			
Female	465 (41.8)	1472 (41.2)	1291 (42.0)
Male	647 (58.2)	2096 (58.8)	1780 (58.0)
Age in years			
15–24	567 (51.0)	1733 (48.6)	1308 (42.6)
25–49	545 (49.0)	1836 (51.4)	1763 (57.4)
Highest education level of women			
No education	259 (23.3)	767 (21.5)	502 (16.3)
Primary education	282 (25.4)	1533 (43.0)	1040 (33.9)
Secondary education	495 (44.5)	728 (20.4)	885 (28.8)
Higher	76 (6.8)	541 (15.1)	644 (21.0)
Region category			
Urban setting	355 (32.0)	879 (24.6)	906 (29.5)
Agrarian	695 (62.5)	2484 (69.6)	2026 (66.0)
Pastoralist	62 (5.5)	206 (5.8)	138 (4.5)
Employment status			
Not employed	634 (57.1)	1501 (42.1)	1267 (41.3)
Formal employee	429 (38.5)	1729 (48.4)	1628 (53.0)
Non_formal employee	49 (4.4)	339 (9.5)	176 (5.7)
Religion			
Orthodox	811 (72.9)	2345 (65.7)	1874 (61.0)
Muslim	169 (15.2)	601 (16.9)	565 (18.4)
Protestant	114 (10.3)	582 (16.3)	609 (19.7)
Tradition/other/catholic	18 (1.6)	40 (1.1)	25 (0.8)
Marital status			
Never married	519 (46.7)	1525 (42.7)	1276 (41.5)
Married/in union	404 (36.3)	1572 (44.1)	1380 (44.9)
Widowed/divorced/separated	189 (17.0)	472 (13.2)	415 (13.6)
Wealth status			
Poor	14 (1.2)	94 (2.6)	1068 (34.8)
Middle	8 (0.8)	37 (1.1)	670 (21.8)
Rich	1090 (98.0)	3438 (96.3)	1333 (43.4)
Contraceptive use			
No	877 (78.9)	2541 (71.2)	2128 (69.3)
Yes	235 (21.1)	1028 (28.8)	943 (30.7)
Source of water			
Not improved	548 (49.2)	1755 (49.2)	653 (21.2)
Improved	564 (50.8)	1814 (50.8)	2418 (78.7)
Cigarette smoking			
No	1110 (99.8)	3562 (99.8)	3057 (99.5)
Yes	2 (0.2)	6 (0.2)	14 (0.5)
Alcohol use			
No	422 (92.8)	1780 (49.9)	1714 (55.8)
Yes	33 (7.2)	1789 (50.1)	1357 (44.2)

The trend of overweight/obesity increased from 14.2% (95% CI 12.3, 16.4) in 2005 to 21.4% (20.1, 23.0) in 2016 (Table 2).

**Table 2:** Trends of overweight and obesity among urban women from 2005 to 2016, Ethiopia

Survey year	Prevalence with 95%CI
2005	14.19 [12.26, 16.37]
2011	14.94 [13.81, 16.15]
2016	21.47 [20.05, 22.96]

The trend in the overweight/obesity rate among urban women within five years before the survey showed variation in characteristics. The overweight/obesity rate increased by 8.8 points for women aged 25 to 49 years from 2005 to 2016. Concerning marital status, the overweight/obesity rate increased in the second phase by 7.6 points for married women and also, in the third phase at an 8.6-point increase. Regarding maternal education, the percentage of overweight/obesity rate increased by 11.1-point percent among primarily educated women from 2005 to 2016. Concerning cigarette smoking, there was a decline in the overweight/obesity rate among women who smoke cigarettes by 25.5 points from 2005 to 2011(Table 3).

**Table 3:** Trend of overweight and obesity among urban women by selected characteristics in 2005, 2011, and 2016, Ethiopia

Characteristics	Survey years			Point difference in overweight/obesity rate		
	EDHS 2005 N = 1112	EDHS 2011 N = 3569	EDHS 2016 N = 3071	Phase 1	Phase 2	Phase 3
				2011–2005	2016–2011	2016–2005
Sex of household head						
Female	14.7	11.3	20.8	− 3.4	9.5	6.1
Male	13.8	17.5	22.0	3.7	4.5	8.2
Age in years						
15–24	7.4	7.4	10.0	0.0	2.6	2.6
25–49	21.2	22.1	30.0	1.0	7.9	8.8
Religion						
Orthodox	14.1	13.6	21.8	− 0.5	8.2	7.7
Muslim	16.6	18.0	22.7	1.4	4.7	6.1
Protestant	12.6	17.7	19.6	5.1	1.9	7.0
Others	7.8	7.7	18.3	− 0.1	10.6	10.5
Marital status						
Never married	8.4	7.4	11.2	− 1.0	3.8	1.8
Married	21.1	22.1	29.7	1.0	7.6	8.6
Widowed/divorced	15.5	15.3	25.6	− 0.2	10.3	10.1
Education status of women						
No	11.5	13.7	18.9	2.2	5.2	7.4
Primary	10.6	13.1	21.7	2.5	8.6	11.1
Secondary	16.9	16.9	19.9	0.0	3.0	3.0
Higher	19.2	19.4	25.3	0.2	5.9	6.1
Employment status of the women						
Not employed	12.0	13.7	17.1	1.7	3.4	5.1
Formal employee	18.5	17.2	26.1	− 1.3	8.9	7.6
Non-formal employee	5.0	9.2	10.0	4.2	0.8	5.0
Wealth index						
Poor	0.0	2.8	9.3	2.8	6.5	9.3
Middle	0.4	0	21.1	− 0.4	21.1	20.7
Rich	14.5	15.4	31.4	0.9	16.0	19.9

### Decomposition analysis

The overall multivariate decomposition analysis (2005 to 2016) revealed that approximately 61.3% of the overall increase in overweight/obesity among urban women was due to the difference in coefficient (difference in the effect of characteristics) across the surveys whereas the remaining was due to the difference in composition of the respondent (endowment) across the surveys (Table 4).

**Table 4:** Overall decomposition analysis of change in overweight and obesity in urban Ethiopia 2005–2016

Overweight/obesity	Coefficient	[95% Interval]	Pct.
E	0.028	[0.016, 0.040]	38.69***
C	0.045	[0.016, 0.073]	61.31*
R	0.072	[0.044, 0.102]	

*E endowment, *C* coefficient, *R* residual; *p value < 0.05; **p value < 0.01

In the detailed decomposition analysis, among the change due to composition (endowment); change in the composition of age in 25–49 years (β = 0.012, 95% CI 0.008, 0.015), married women (β = 0.010, 95% CI 0.006, 0.014), widowed/divorced women (β = − 0.002, 95% CI 0.005, − 0.001), women with primary (β = 0.007, 95% CI 0.003, 0.011), secondary (B = − 0.016, 95% CI − 0.023, − 0.007), and higher education (β = 0.014, 95% CI 0.006, 0.022), formal employment(β = 0.006, 95% CI 0.001, 0.011), and informal employment status (β = − 0.002, 95% CI − 0.003, − 0.0004) significantly contribute to overweight/obesity rate positively over the last 11 years (from 2005 to 2016). However, there are no significant factors attributed to the difference in coefficients for overweight/obesity rate (Table 5).

**Table 5:** Detailed decomposition analysis of change in overweight and obesity in urban Ethiopia 2005–2016

Overweight/obesity	Difference due to characteristics(E)		Difference due to coefficient (C)	
	Coefficient [95%CI]	Percent	Coefficient [95%CI]	Percent
Age in years				
15–24				
25–49	0.012 **[0.008, 0.015]	16.38	0.009 [− 0.034, 0.052]	12.65
Marital status				
Not married	0.010 ***[0.006, 0.014]	13.81	0.012 [− 0.025, 0.050]	17.07
Married				
Widowed/divorced	− 0.002 *[− 0.005, − 0.001]	− 3.48	0.002 [− 0.017, 0.020]	2.44
Religion				
Orthodox				
Muslim	0.0006 [− 0.001, 0.002]	0.86	− 0.006 [− 0.019, 0.006]	− 8.49
Protestant	− 0.0007 [− 0.006, 0.004]	− 0.96	− 0.0006[− 0.012, 0.011]	− 0.83
Others	0.0005 [− 0.001, 0.002]	0.77	0.001 [− 0.004, 0.005]	1.03
Educational status				
No				
Primary	0.007 [0.003, 0.011] *	9.80	0.011 [− 0.019, 0.042]	15.55
Secondary	− 0.016 [− 0.023, − 0.007] ***	− 21.39	− 0.005 [− 0.051, 0.040]	− 6.75
Higher	0.014 [0.006, 0.022] **	19.94	− 0.0004 [− 0.009, 0.008]	− 0.53
Employment status				
Not employed				
Formal	0.006 [0.001, 0.011] *	8.75	0.006 [− 0.021, 0.031]	7.66
Informal	− 0.002 [− 0.003, − 0.0004] *	− 2.30	0.001 [− 0.008, 0.008]	0.68
Contraceptive use				
No				
Yes	− 0.002 [− 0.007, 0.001]	− 3.46	− 0.008 [− 0.027, 0.009]	− 12.25
Cigarette smoking				
No				
Yes	− 0.00001[− 0.001, 0.0005]	− 0.022	− 0.0004 [− 0.001, 0.0001]	− 0.55
Constant	0.024 [− 0.097, 0.146]	33.63		

Key: ***significant at *p* value of 0.001; **significant at *p* value of 0.01; *significant at *p* value of 0.05

### Variations in overweight/obesity due to differences in the age of women

**Fig. 1:**
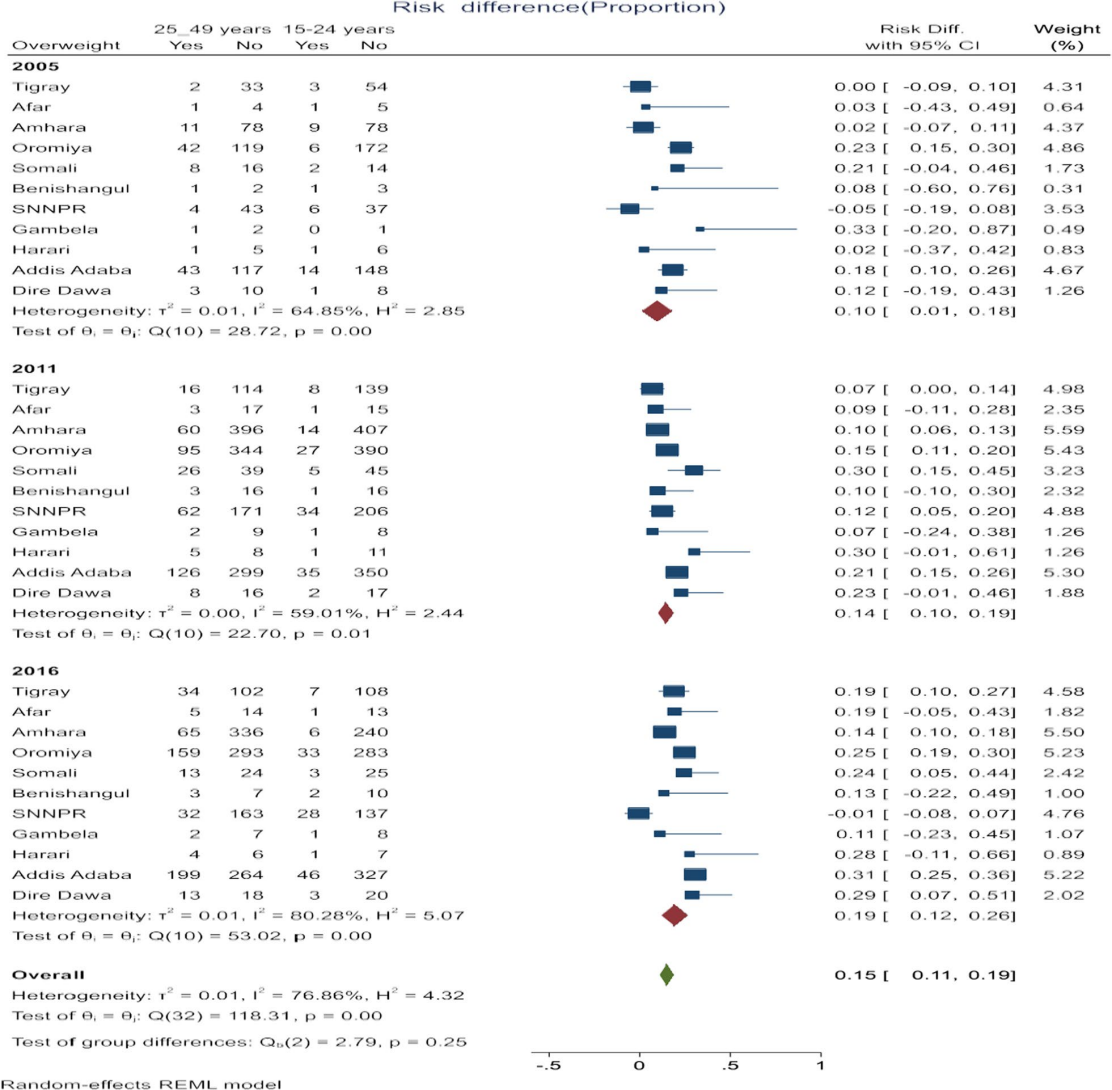
Forest plot of risk difference between women from the age group below 25 and above 25 years in overweight/obesity across regions in Ethiopia from 2005 to 2016

The risk difference (RD) in women’s overweight/obesity significantly varied across regions in urban Ethiopia during each survey (EDHS 2005, 2011, and 2016). In EDHS 2005, overall, there was a significant risk difference in overweight/obesity with an age difference (RD = 0.15, 95% CI 0.11, 0.19). The highest significant age difference in overweight/obesity was observed in Addis Ababa city which was (RD = 0.31, 95%: 0.25, 0.36) followed by the Somali region (RD = 0.30, 95% CI 0.15, 0.45). In EDHS 2011, overall, there was a significant age difference in overweight/obesity in urban Ethiopia (RD = 0.10, 95% CI 0.01, 0.18). The highest significant risk difference was observed in the Oromia region (RD = 0.23, 95% CI 0.15, 0.30). In EDHS 2016, there was a significant risk difference in overweight/obesity between women who were in the age group of 15–24 years and 25–49 years across regions in urban Ethiopia (RD = 0.14, 95% CI 0.10, 0.19). The highest significant age difference in overweight/obesity was observed in Addis Ababa city (RD = 0.31, 95% CI 0.25, 0.36) while the lowest risk difference in the Amhara region (RD = 0.14, 95% CI 0.10, 0.18). Generally, the risk difference among the three surveys year in overweight/obesity between women who were in the age group of 15–24 years and 25–49 years across regions in urban Ethiopia significantly increased as survey years (RD = 0.10 in 2005, RD = 0.14 in 2016 and RD = 0.19 in 2016) (Fig. 1).

### Spatial distribution of overweight/obesity in three consecutive surveys

**Fig. 2:**
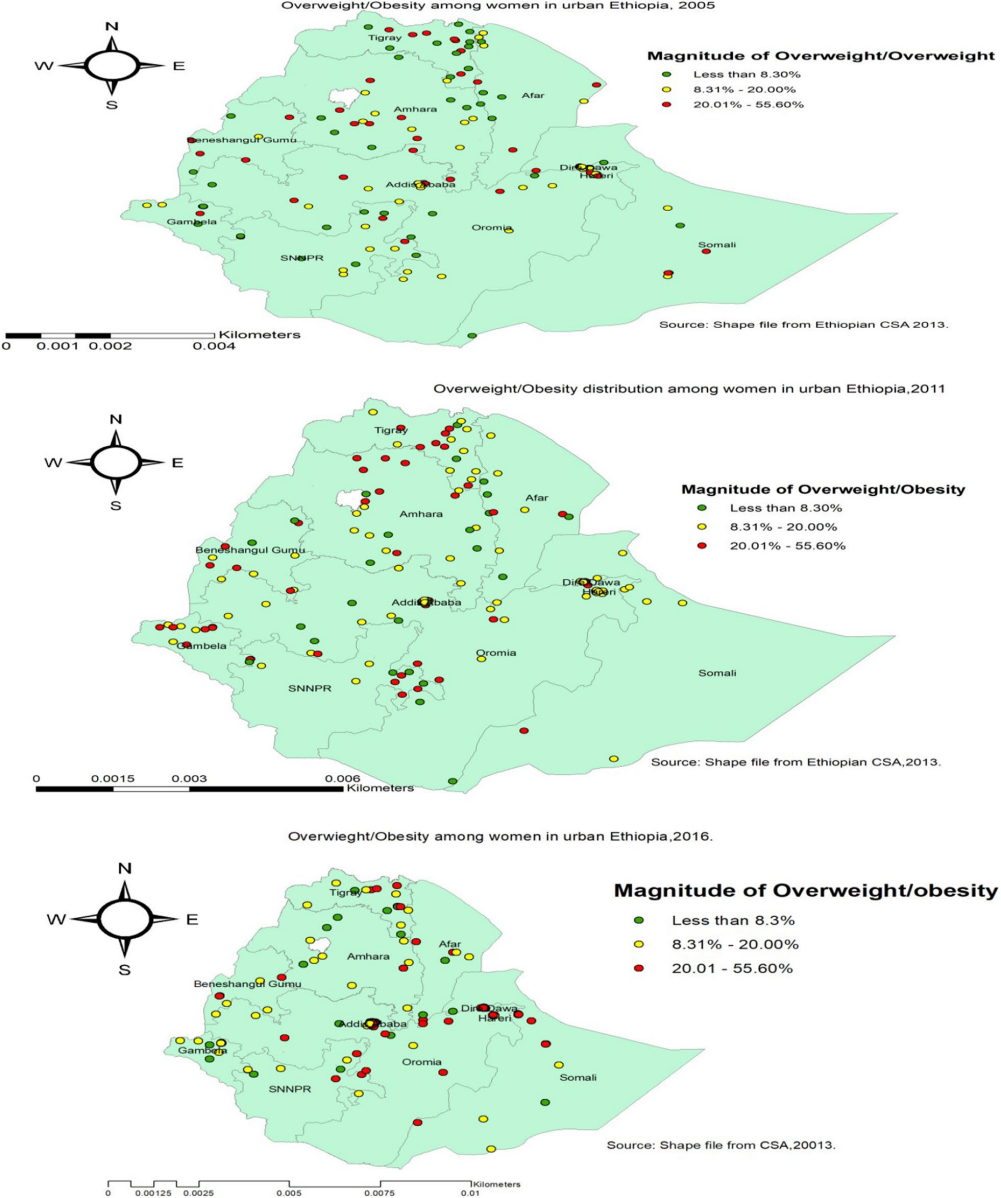
Spatial distribution of overweight or obesity among urban women using the three consecutive survey years, (2005, 2011 and 2016)

The spatial distribution of overweight/obesity in urban Ethiopia was non-random among the three consecutive surveys. The global Moran’s I value was 0.12 (*P* value < 0.001) in 2005, 0.59 (*P* value < 0.001) in 2011, and 0.44 (*P* value < 0.001) in 2016 Ethiopian Demographic and health surveys. The spatial distribution of Overweight/obesity in urban Ethiopia was different in the three survey years. In EDHS 2005, a high proportion of overweight/obesity was found mainly in Tigray, Amhara, and Addis Ababa. In EDHS 2011, high clustering of overweight was detected in most parts of Tigray, North Amhara, and the borderline of Oromia and SNNPR regions of Ethiopia. Besides, a high proportion of overweight/obesity was detected in the Northeast parts of Tigray, Southern Oromia, Dire Dawa, Harari, and Northern Somali region in EDHS 2016 (Fig. 2).

**Fig. 3:**
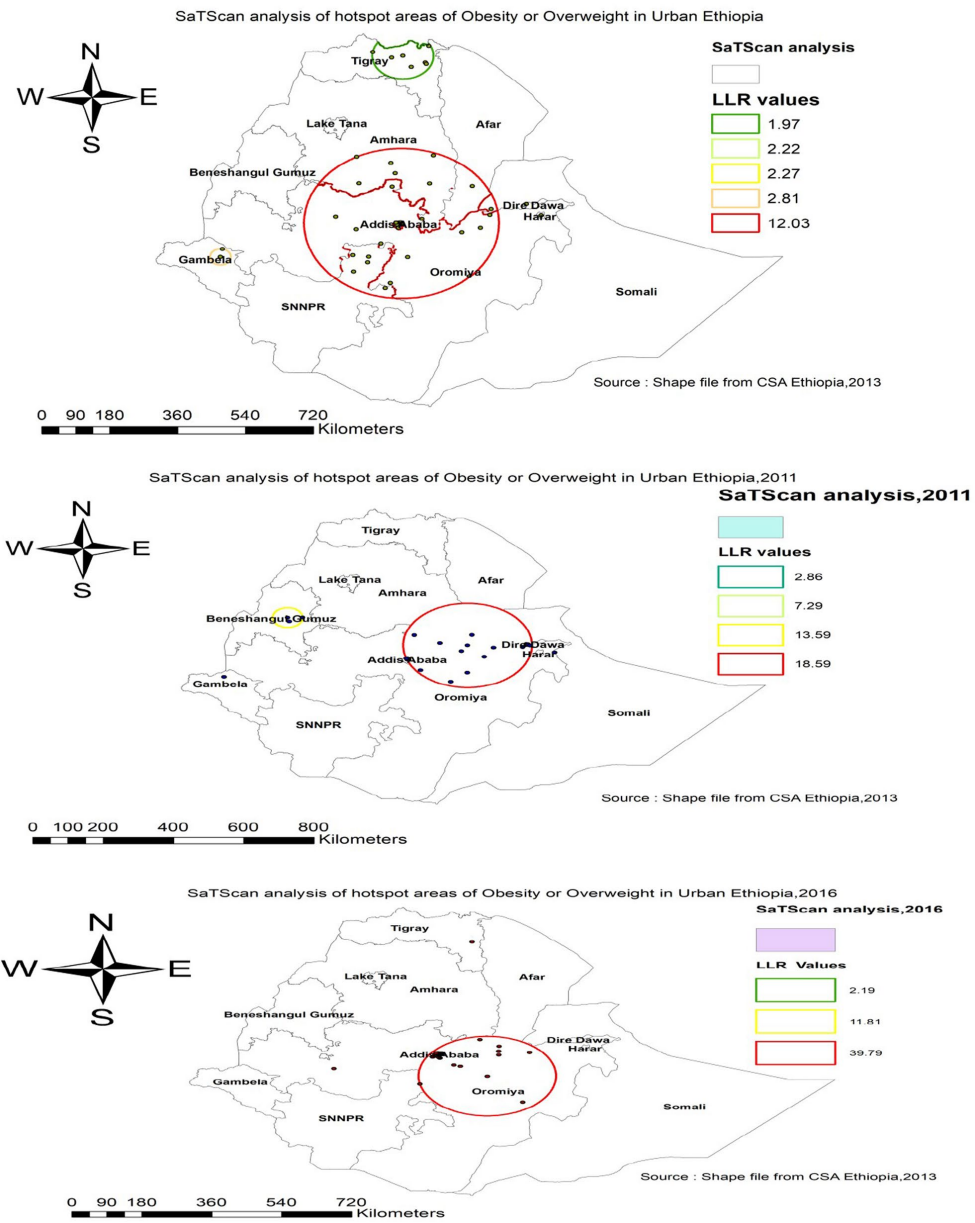
SaT Scan analysis of overweight or obesity among urban women using the three consecutive survey years, (2005, 2011 and 2016)

In EDHS 2005, the spatial scan statistics identified a total of 36 primary and secondary clusters of overweight/obesity. Of these, 34 clusters were most likely (primary cluster), the spatial window was located in southern Amhara, Addis Ababa, and Northwest Oromia regions centered at 9.030245 N, 38.857304 E with 255.37 km, a Relative Risk (RR) of 2.22, and Log-Likelihood (LLR) of 12.03, at *p* value < 0.01 (Table 6). It showed that women within the spatial window had a 2.22 times higher likelihood of being overweight/obese as compared to women outside the spatial window. Whereas the secondary clusters were located in the Tigray regions (Fig. 3).

**Table 6:** SaT Scan analysis of overweight/obesity among women in rural Ethiopia, EDHS 2005, 2011 and 2016

Survey year	Cluster type	Significant enumeration areas (clusters) detected	Coordinates/radius	Populations	Cases	RR	LLR	*P* value
2005	Primary	402, 287, 155, 19, 293, 451, 302,369, 91, 236, 121, 139, 438, 23,510, 359, 310, 271, 122, 174, 10,399, 531, 391, 331, 476, 176,308, 135, 333, 491, 18, 452, 24	*(9.030245 N, 38.857304 E)/255.37 km*	252	58	2.22	12.03	≤ 0.001
	Secondary	265, 221	*(8.001711 N, 34.537071 E) /27.39 km*	21	7	2.58	2.81	0.890
2011	Primary	564, 230, 336, 506, 491, 201,	*9.555410 N, 40.326164 E) /180.77 km*	470	113	1.89	18.56	≤ 0.001
	Secondary	137, 244, 36	*(10.637520 N,35.719208 E) /42.00 km*	84	31	2.69	13.59	≤ 0.001
2016	Primary	319, 149, 290, 71, 49, 230, 353, 83, 286, 236, 252, 402, 412, 90,211,330, 539, 451, 560, 475,261, 509, 155, 287, 428, 264, 61,19,293, 225, 302, 639, 110, 247,159, 15, 414, 582, 153, 464, 305,635, 170, 195, 59, 645, 108, 314,144, 608, 487, 624, 626, 100,145, 31,369, 112, 532, 91, 107,147, 339, 463, 11, 274, 14	*(8.152924 N, 39.893330 E)/175.24 km*	1324	381	1.86	39.79	≤ 0.001
	Secondary	411	*(8.453819 N,36.343666 E 0 km*	174	64	1.82	11.81	0.001

In EDHS 2011, the spatial scan statistics identified a total of 35 primary and secondary clusters of overweight or obesity. Of these, 32 clusters were most likely clusters, which were located in Addis Ababa, Harari, and Dire Dawa city, centered at 9.555410 N, 40.326164 E with 180.77 km radius, a Relative Risk (RR) 1.89, and Log-Likelihood Ratio (LRR) of 18.56, at p-value < 0.01 (Table 6). It showed that women within the spatial window had a 1.89 times higher likelihood of being overweight/obese as compared to women outside the spatial window. Whereas the secondary clusters were located in the central Benishangul region (Fig. 3).

In EDHS 2016, the SaTScan statistics identified a total of 64 primary and secondary clusters of these, of these 63 were most likely clusters which were located in Addis Ababa, and the north Oromia region centered at 8.152924 N, 39.893330 E with a 175.24 km radius, RR of 1.86 and LLR of 39.79, at *p* value < 0.01 (Table 6). It showed that women within the spatial window had a 1.86 times higher likelihood of overweight/obesity as compared to women outside the spatial window (Fig. 3). Overall, the SaTScan analysis revealed that Addis Ababa and the Oromia region were persistently at higher risk of overweight/obesity across the three surveys.

**Fig. 4:**
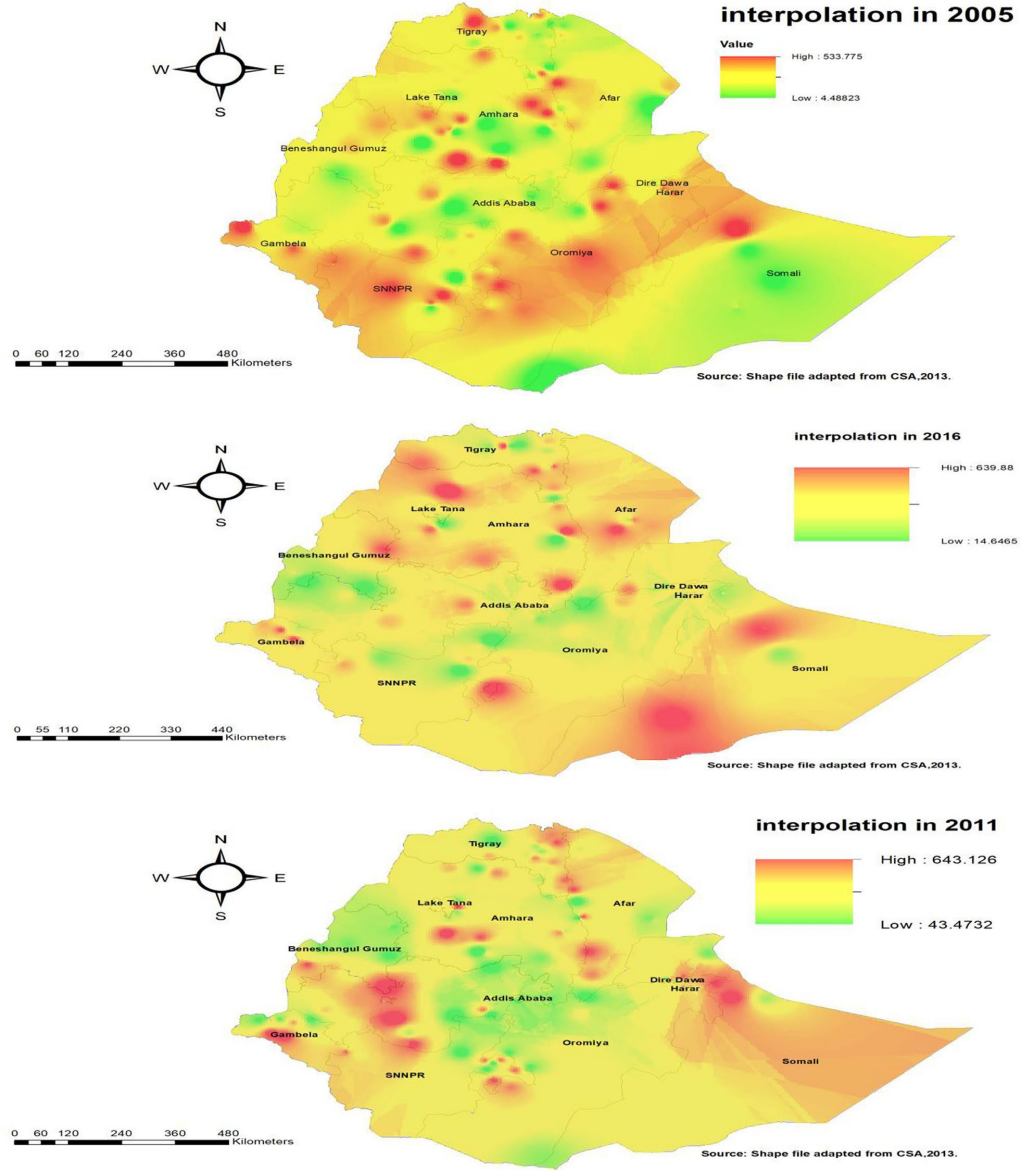
Spatial interpolation of overweight among urban women using the three consecutive survey years, (2005, 2011 and 2016)

Kriging interpolation of overweight/obesity based on EDHS 2005, Kriging interpolation predicts that the highest overweight/obesity was detected in the northern part of Somali, central Oromia, Northern SNNPR, and Harari regions whereas, predicted relatively low overweight/obesity located in the Benishangul Gumuz, Afar and Amhara regions (Fig. 4). In 2011, Kriging interpolation revealed that the highest predicted prevalence of overweight/obesity was found in Oromia, Amhara, and Somali regions. In contrast, predicted low overweight/obesity were detected in Tigray, Benishangul Gumuz, and SNNPR regions (Fig. 4). From EDHS 2016 data, Kriging interpolation predicted that east Somali, Southern Amhara, and central SNNPR had the highest overweight/obesity while Benishangul Gumuz and Oromia regions contained relatively low overweight/obesity (Fig. 4).

## Discussion

Overweight/ obesity among women is a major health challenge [32]. The current study focused only on urban women since there was a remarkable nutritional transition during the past two recent decades. Urbanization was highly related to different dietary and behavioral risk factors and subsequently increases overweight/obesity [33, 34]. On the other hand, changes in lifestyles may result in lower physical activities in urban women [35]. Based on the aforementioned statement, we assessed the change in overweight/obesity in urban women of Ethiopia for the last two decades. The trends of overweight/obesity among urban women increased from 14.2% in 2005 to 21.4% in 2016 with an annual rate of increment of 1%. This finding showed a slight decline in overweight/obesity compared with a study done in other urban African countries [36] which was the prevalence of overweight/obesity increased by an average of nearly 5% per year.

About 61.3% of the overall increment of overweight/obesity was attributed to the change in the composition of the respondents and the remaining 38.7% of the overall increase in overweight/obesity was due to the change in the effects of explanatory variables (coefficients). This might be due to the population structure changes such as increased literacy levels and improvement of socio-demographic and economic characteristics that contributed to the increment of overweight/obesity among urban women in Ethiopia [37].

There was a significant risk difference for overweight/obesity in ages between 15–24 and 15–49 years across regions over the three surveys. An increase in the composition of women in the age group of 25–49 years showed a positive relationship with overweight/obesity. This is in agreement with other studies conducted in Zimbabwe, Bangladesh, and Maldives [3, 7, 35]. The 25–49 years is a reproductive peak age for women characterized by increased parity, contraceptive use, and related changes in body composition which entails a higher probability of overweight/obesity [3, 38]. Moreover, these finding also supports the study done in Morocco [39] which tells an age effect in the development of obesity. The reason for an increment in overweight or obesity in older age might be the fat begins to accumulate early in young women and, with increasing age, it is redistributed to the abdominal cavity that suggesting a high health risk for these women [40].

There was a positive change in overweight/obesity as the proportion of women who attended secondary and higher levels of education increased. This is consistent with other studies conducted in different countries [2, 10–12]. The possible explanation is women with a higher level of education tend to be employed in white-collar jobs and are more likely to have a sedentary life. Additionally, these women may have higher incomes and might be exposed to an energy-dense westernized diet [13]. On the other hand, the current study disagrees with a study done in Brazil [6]. This difference might be the difference in the study population which included both men and women, residents, with the age group 20–60 years[41].

The other remarkable finding in this part of the analysis was the effect of marital status. Overweight/obesity among urban reproductive age group women for the last 10 years was different in the marital status which is due to the change in the proportion of married women. An increase in overweight/obesity was due to a change in the proportion of women who were married compared to single women. This might be because when women became married, they will have a stable way of life which leads to an increase in overweight/obesity. Besides, this might be explained in other words married women unlike single are more likely to be multiparous, so they are at greater risk of weight gain during pregnancy and the puerperium period [26]. Furthermore, married people might not think more about keeping a healthy weight to attract potential mates. Married people have also less time for physical activity since they have more family duties. After getting married, their eating habits are more likely to change, and their commitment to maintaining a healthy weight may decrease [42, 43].

The last remarkable finding in this part of the analysis was the effect of employment status. The higher proportion of women with formal employment in the governmental organization changed overweight/obesity in a positive direction. This finding agreed with another study [36]. The higher overweight/obesity among formally employed women might be associated with the change in nutritional and lifestyle trends that have fewer opportunities for physical activities. Moreover, the possible reason for differences in overweight/obesity between women who had formal work and no work might be in formal work, women mostly not engaged in laborious activities and therefore, are likely to gain as much weight as women with no work. On the other hand, a decrease in overweight/obesity was due to a change in the proportion of women who had informal employment compared to no work. This might be because women with informal employment had a higher opportunity for physical activity subsequently decreasing overweight/obese [35]. The other possible justification is related to working hours. Formally employed women who worked for ≥ 60 h. per week is more likely to experience obesity. Obesity rates also increase among female workers with longer working hours [44].

Tigray region and Addis Ababa city administration had a high proportion of overweight/obesity consistently in three consecutive EDHS years. On the other hand, overall, the SaTScan analysis revealed that Addis Ababa and Oromia regions were persistently at a higher risk of overweight/obesity across the three surveys. Kriging interpolation predicts that the highest overweight/obesity was detected in the Oromia, Amhara, and Somali regions.

## Strengths and limitations of the study

To the best of our knowledge, this study is the first of its kind that identifies the trend contributions of factors to the change in overweight/obesity in urban Ethiopia. The study utilized a large dataset representing the whole urban side of the country, Ethiopia. Large sample size with weighting the data for the sampling probability and non-response to make the data nationally represented. Complex sampling procedures were also considered during the testing of statistical significance. However, since the data was collected based on self-report from mothers, this could make the data prone to recall and social desirability bias.

## Conclusions

The rate of overweight/obesity among urban women of the reproductive age group in Ethiopia has shown a significant increment over the last 11 years’ time period. The majority of the overall change in overweight/obesity over the study period was attributable to the change in coefficients of selected explanatory variables between 2005 and 2016.

The change in the composition of women’s age, educational status, marital status, and employment status indicated a significant effect on the change in overweight/obesity. Program interventions should be targeted in urban areas that had higher overweight/obesity, especially for some categories of the population, including older (above 25 years), educated, married, Addis Ababa residents, and formally employed women.

## Data Availability

The data sets used during the current study are available from the corresponding author on a reasonable quest.

## Abbreviations

ARR: Annual Rate of Reduction
BMI: Body Mass Index
DHS: Demographic Health Survey
EAs: Enumeration areas
EDHS: Ethiopian Demographic and Health Survey
PHC: Population and Health Survey
RD: Risk difference
WHO: World Health Organization
